# Ambient AI Scribes and Emergency Department Documentation Burden: Retrospective Cohort Study

**DOI:** 10.2196/92193

**Published:** 2026-07-02

**Authors:** Carl Preiksaitis, Al'ai Alvarez, Maia Winkel, Mia Karamatsu, Ian Brown, Neetha Sama, Luke Morris, Jae-yeon Lee, Allie Gubbels, Eileen Wahl, Anna Frye, Austin Schoeffler, Laleh Gharahbaghian, Christian Rose

**Affiliations:** 1 Department of Emergency Medicine School of Medicine Stanford University Palo Alto, CA United States; 2 Stanford Health Care Palo Alto, CA United States

**Keywords:** artificial intelligence, AI, ambient scribes, documentation burden, electronic health records, emergency medicine, physician burnout, clinical informatics, workflow efficiency

## Abstract

**Background:**

Clinician burnout has reached crisis levels in emergency medicine, with clinical documentation burden identified as a central contributing factor. Ambient artificial intelligence (AI) scribes offer a promising approach to reduce this burden, but objective evidence in the emergency department (ED) setting remains limited, and prior reports have been constrained by short observation windows and low adoption.

**Objective:**

This study aimed to evaluate the association between ambient AI scribe use and on-shift documentation time during a 13-month staged rollout in a busy ED, accounting for physician- and patient-level factors.

**Methods:**

We conducted a retrospective cohort study at a tertiary academic ED from February 2025 to March 2026. The analytic cohort comprised 10,344 encounters managed by 100 attending physicians across 4 ED care settings. We restricted analysis to encounters managed by a single attending physician and excluded those with human scribes. The comparison group comprised encounters in which the ambient AI scribe was not used; use was determined entirely at attending physician discretion on an encounter-by-encounter basis. The primary outcome was on-shift documentation time derived from electronic health record audit logs. We used mixed-effects linear models with physician random intercepts to adjust for patient and encounter characteristics.

**Results:**

Ambient AI scribe use was associated with a 72.6-second reduction in on-shift documentation time per encounter (95% CI 63.8-81.4; *P*<.001). The effect was similar in magnitude for high-use physicians (use rates of ≥18.2%, which was the cohort mean; −71.6 seconds) and low or moderate users (−64.2 seconds), with no statistically significant difference (*P*=.51). Note character count decreased by 690 characters (95% CI 273-1107; *P*=.001); after-shift documentation time increased modestly by 9.1 seconds (95% CI 2.9-15.3; *P*=.004). Negative control outcomes were largely null, and a within-clinician placebo permutation test yielded a distribution centered at 0 (mean −0.8 seconds), inconsistent with the observed effect arising from confounding alone.

**Conclusions:**

In this single-center analysis, ambient AI scribe use was associated with a statistically significant reduction in on-shift documentation time (*P*<.001), equivalent to approximately 24 minutes per 8-hour shift if used across 20 encounters. These findings extend prior descriptive work with adjusted inferential evidence and support the clinical relevance of ambient AI scribes for ED documentation burden, although the magnitude of benefit varies by physician, patient, and workflow factors.

## Introduction

Clinician burnout has reached crisis levels in emergency medicine, with clinical documentation burden identified as a central contributing factor to workflow inefficiency and job dissatisfaction [1,2]. One survey of nearly 1400 emergency physicians found that 31% self-reported spending more than 6 hours per week on after-hours charting [3]. In the emergency department (ED), high patient acuity, rapid turnover, constant task switching, and 24/7 clinical operations amplify this burden. Objective electronic health record (EHR) audit log studies show that documentation constitutes a substantial portion of clinician time per encounter and varies by factors such as acuity, admission status, and handoffs, underscoring the importance of contextual determinants of documentation effort [4,5]. In this environment, even modest per-encounter efficiencies can compound into meaningful improvements in clinician well-being and throughput.

Ambient artificial intelligence (AI) scribes, which are systems that passively capture the physician-patient dialogue and generate draft notes, offer a promising approach to reduce documentation burden while prioritizing physician attention to the patient [6-8]. We frame these tools through a sociotechnical lens: their effectiveness depends not only on the underlying model’s intrinsic capability but on contextual fit with task complexity, the operational environment, and individual user adoption patterns [9]. Existing studies, predominantly in outpatient and primary care settings, document subjective well-being benefits but yield mixed evidence on objective time savings, leaving uncertainty about ambient AI scribes as a validated intervention for documentation burden [10-14]. Despite rapid health system adoption, there remains a critical evidence gap in ED-focused analyses that use objective audit log outcomes and adjust for ED-specific factors such as patient acuity, complexity, zone-specific workflows, and individual physician variation in documentation practices [4,15].

In a recent descriptive analysis from our institution covering the first 8 months of ED rollout, we characterized early adoption patterns of ambient AI scribes, finding low but highly skewed uptake (11.2% of encounters; 38% of attending physicians) clustered in low-acuity zones, with unadjusted median documentation time approximately 65 seconds shorter for ambient encounters [16]. This study extends this work. It draws on a 13-month observation window during which adoption matured; uses mixed-effects models accounting for physician-level clustering and patient and encounter characteristics; and incorporates negative control, placebo permutation, sensitivity, and doubly robust analyses to assess the robustness of the observed associations.

The goal of this investigation was to quantify the association between ambient AI scribe use and objective documentation burden in the ED. Our primary objective was to estimate the change in on-shift documentation time after adjustment for the operational complexities of the ED environment, including workflow variations across clinical zones, patient acuity, and individual physician documentation habits. Specifically, we asked the following: Among adult ED encounters managed by a single attending physician in core clinical zones, is the use of an ambient AI scribe associated with a reduction in on-shift documentation time after adjusting for patient acuity, complexity, clinical zone, and physician-level variation? We aimed to provide objective, ED-specific evidence to inform implementation decisions for a technology now being broadly deployed across health systems.

## Methods

### Study Design and Setting

We conducted a retrospective cohort study at a high-volume tertiary academic ED analyzing data from February 1, 2025, to March 1, 2026. We adhered to the STROBE (Strengthening the Reporting of Observational Studies in Epidemiology) guidelines and to recent guidance for reporting analyses of EHR metadata [17].

### Selection of Participants

The source population included all adult (≥18 years) ED encounters during the study period. To create a direct comparison between attending physicians using traditional documentation methods and those using an ambient AI scribe, we restricted the analysis to encounters where documentation was performed exclusively by attending physicians. Due to shared documentation practices at our institution, we additionally restricted our analysis to encounters where care was administered by a single attending physician (no care handoffs). We excluded encounters primarily managed in the resuscitation and pediatric zones due to distinct workflows and documentation practices in those areas. This was a complete-case cohort drawn from all eligible encounters during the study window; no random sampling was performed. The denominator, therefore, represents the full population of ED encounters meeting the inclusion criteria during the staged rollout.

### Intervention

Stanford Health Care collaborated with Epic Systems Corporation and Nuance Communications to integrate Microsoft DAX Copilot ambient AI scribe technology into clinical workflows. The tool enabled clinicians to record patient-clinician conversations directly from the Epic Haiku mobile app. Draft note content was parsed into 4 auto-populated Epic SmartSections (history of present illness, physical examination, results, and assessment and plan) that could be embedded into existing note templates alongside other documentation tools. AI-generated content was automatically populated within minutes of concluding the recording. An attestation SmartSection documented patient notification and clinician review. Training resources, including knowledge base articles, presentations, and individual or group sessions, were available to physicians. The system was made available through a staged rollout during the study period, with voluntary adoption based on individual preference.

Use of the ambient AI scribe was determined entirely at attending physician discretion on an encounter-by-encounter basis; no encounter-level criteria, prompts, or assignments influenced use. The comparison group, therefore, comprised all eligible encounters in which the attending physician did not use the ambient AI scribe, including both encounters managed by attending physicians who never adopted the tool and encounters in which adopting attending physicians opted to document conventionally. This design contrasts ambient encounters against a fully uncontrolled comparator and motivated the adjustment, sensitivity, and falsification analyses described below.

### Measurements

Data were extracted from Epic EHR systems using 2 primary data sources: Epic’s User Action Log (UAL Lite), which provides developer-derived measures of user activity duration, and Epic’s *note_attribution* table, which contains clinical documentation metadata, including character counts, entry methods, and attribution data [17]. UAL Lite provides measures of time that users spend on specific EHR activities aggregated hourly, calculated using Epic’s standard methodology with a 5-second time-out threshold for inactivity. Individual user interactions within each hour are aggregated by Epic’s algorithms to produce total active time per activity category. *Notes* activities were defined using an established categorization schema and mapped from Epic’s raw activity codes to clinically meaningful documentation tasks [4]. Attending physician shift schedules were obtained from clinical scheduling systems to establish temporal boundaries. Activity times were aggregated at the patient encounter level (normalization denominator) and classified as either on shift (during scheduled shift hours) or after shift (occurring any time after shift end) based on scheduled work periods.

Ambient encounters were identified as those with nonzero ambient AI-generated character counts in the *note_attribution* table. Covariates included patient age; number of ED diagnoses; and categorical indicators for Emergency Severity Index (ESI) level and clinical zone area, reflecting 4 distinct workflows: main ED (stretcher-based rooms for nonresuscitation care), vertical care (chair-based care for ambulatory patients), triage and lobby (rapid assessment area), and telemedicine (on-site patients managed via video by a telemedicine ED physician).

### Outcomes

The primary outcome was on-shift documentation time measured in seconds, defined as time spent in EHR activities contributing to clinical note generation. Secondary outcomes included total attending physician EHR time per encounter, after-shift documentation time, and note character count.

### Analysis

Descriptive statistics were calculated as frequencies for categorical variables and medians and IQRs for continuous variables. Continuous outcomes showed right-skewed distributions (skewness=1.51-4.54). We used linear mixed-effects models for inferential analysis, which are robust with large samples. The intraclass correlation coefficient was 0.39, indicating that 39% of the variance in documentation time was attributable to physicians, necessitating mixed-effects modeling [18]. Model assumptions were checked via residual plots; variance inflation factors of less than 2 confirmed no multicollinearity.

The primary model was a linear mixed-effects model with a random intercept for attending physician evaluating the association between ambient AI scribe use and on-shift documentation time. Fixed effects included patient age, number of ED diagnoses, and categorical variables for ESI and clinical zone group, as well as zone-by-acuity interaction terms to account for differential baseline documentation patterns. ESI level 1 encounters (8/10,344, 0.1% of the cohort) were excluded from the primary model due to data sparsity (no ESI level 1 encounters used ambient scribes) and distinct care patterns. In a sensitivity analysis, we reran the model including ESI level 1 encounters using main effects only; the results were substantively unchanged (coefficient=−72.4 seconds, 95% CI −81.2 to −63.6; *P*<.001).

To assess heterogeneity of effect, we tested for interactions between ambient AI scribe use and both physician use level (high vs low or moderate) and clinical zone group. We prespecified high-use physicians as those with ambient AI scribe use rates at or above the cohort mean of 18.2%. We also modeled a continuous dose-response relationship between the proportion of AI scribe–generated note content and documentation time.

Because the comparison was uncontrolled and physicians may have selectively used ambient AI for encounters they anticipated would be straightforward in ways our covariates did not capture, we conducted 3 classes of analyses targeted at residual confounding. First, we evaluated negative control outcomes: 4 EHR activity measures unrelated to note documentation (on-shift order entry time, on-shift in-basket time, after-shift in-basket time, and after-shift chart review time). If our primary effects were driven by the selection of less complex encounters rather than by the tool itself, we would expect spillover into these activities. Second, we conducted a within-clinician placebo permutation test in which the ambient use indicator was randomly shuffled across each clinician’s own encounter set 200 times, generating a null distribution of effect estimates against which the observed effect was compared. Third, we conducted sensitivity analyses including log transformation, gamma generalized estimating equations, temporal trend adjustments (linear time trend and week fixed effects), and doubly robust estimation [19]. We also reran the primary model adjusting for additional patient demographics (sex, race, ethnicity, language, and whether an interpreter was needed); the effect estimate was effectively unchanged (−71.7 seconds vs −72.6 seconds; Multimedia Appendix 1). Technical details are provided in Multimedia Appendix 1.

All analyses were performed using Python (version 3.11; Python Software Foundation, Wilmington, DE) and the *pandas* and *statsmodels* packages. Significance was set at *P*<.05.

### Ethical Considerations

This study was reviewed and approved by the Stanford University Institutional Review Board (protocol 69107), which granted a waiver of informed consent given the retrospective design and the use of existing operational EHR data. No participants were prospectively enrolled, and no compensation was provided. All data were extracted from institutional EHR systems, stored on secure servers managed by the institution with access restricted to the study team, and analyzed and reported only in aggregate form such that no individual patient or clinician can be identified. The study was conducted in accordance with the ethical standards of the responsible institutional committee on human experimentation and with the World Medical Association Declaration of Helsinki.

## Results

### Characteristics of the Study Participants

The final analytic cohort comprised 10,344 encounters managed by 100 attending physicians. Ambient AI scribing was used in 18.2% (1881/10,344) of these encounters. Most patients had an ESI acuity level of 3 (5632/10,344, 54.4%) or 4 (3345/10,344, 32.3%), and 85% (8788/10,344) of the encounters resulted in discharge from the ED. All key variables had complete data with no missing values. Baseline patient characteristics and unadjusted outcomes are shown in Table 1. Encounters with AI scribe use differed from those without on several baseline characteristics: patients were younger (median age 38, IQR 26-55 years vs 41, IQR 28-60 years), had lower acuity (119/1881, 6.3% vs 923/8463, 10.9% ESI level 2), were more often seen via telemedicine (565/1881, 30% vs 859/8463, 10.2%), and had higher discharge rates (1686/1881, 89.6% vs 7102/8463, 83.9%).

**Table 1:** Baseline patient and encounter characteristics^a^.

Characteristics				No ambient scribe (n=8463)		With ambient scribe (n=1881)
**Patient demographics**						
	Age (y), median (IQR)		41 (28-60)		38 (26-55)	
	**Sex, n (%)**					
		Female	4556 (53.8)		1049 (55.8)	
		Male	3904 (46.1)		832 (44.2)	
	**Language, n (%)**					
		English	6450 (76.2)		1470 (78.1)	
		Spanish	1589 (18.8)		335 (17.8)	
		Other	424 (5)		76 (4)	
	Interpreter needed, n (%)		1818 (21.5)		369 (19.6)	
**ESI^b^, n (%)**						
	1 (highest acuity)		8 (0.1)		0 (0)	
	2		923 (10.9)		119 (6.3)	
	3		4585 (54.2)		1047 (55.7)	
	4		2691 (31.8)		654 (34.8)	
	5		256 (3)		61 (3.2)	
**Disposition, n (%)**						
	Discharge		7102 (83.9)		1686 (89.6)	
	Admission		1266 (15)		183 (9.7)	
	Transfer		52 (0.6)		1 (0.1)	
	Other		43 (0.5)		11 (0.6)	
**Means of arrival, n (%)**						
	Self-arrival		7773 (91.8)		1810 (96.2)	
	Ambulance		682 (8.1)		70 (3.7)	
	Other		8 (0.1)		1 (0.1)	
**Chief concern, n (%)**						
	Abdominal pain		509 (6)		129 (6.9)	
	Chest pain		465 (5.5)		121 (6.4)	
	Back pain		300 (3.5)		93 (4.9)	
	Headache		260 (3.1)		43 (2.3)	
	Cough		198 (2.3)		56 (3)	
	Fall		251 (3)		36 (1.9)	
	Other		6231 (73.6)		1332 (70.8)	
Number of ED^c^ diagnoses, median (IQR)				1 (1-2)		1 (1-1)
**ED care area, n (%)**						
	Main ED		2398 (28.3)		256 (13.6)	
	Vertical care		2205 (26.1)		720 (38.3)	
	Triage and lobby		3001 (35.5)		340 (18.1)	
	Telemedicine		859 (10.2)		565 (30)	
**Outcomes (unadjusted), median (IQR)**						
	On-shift documentation time (s)		215 (133-338)		152 (76-242)	
	After-shift documentation time (s)		0 (0-9)		0 (0-0)	
	Total EHR^d^ time (s)		527 (354-786)		471 (310-680)	
	Note characters		8874 (5650-13,598)		8628 (5429-11,929)	

^a^A complete demographic breakdown including all languages and detailed race and ethnicity categories is available in Multimedia Appendix 1.

^b^ESI: Emergency Severity Index. ESI level 1 encounters (8/10,344, 0.1% of the cohort) were excluded from the primary mixed-effects model due to data sparsity and distinct care patterns; a sensitivity analysis including ESI level 1 showed substantively unchanged results.

^c^ED: emergency department.

^d^EHR: electronic health record.

### Primary Outcome

**Figure 1:**
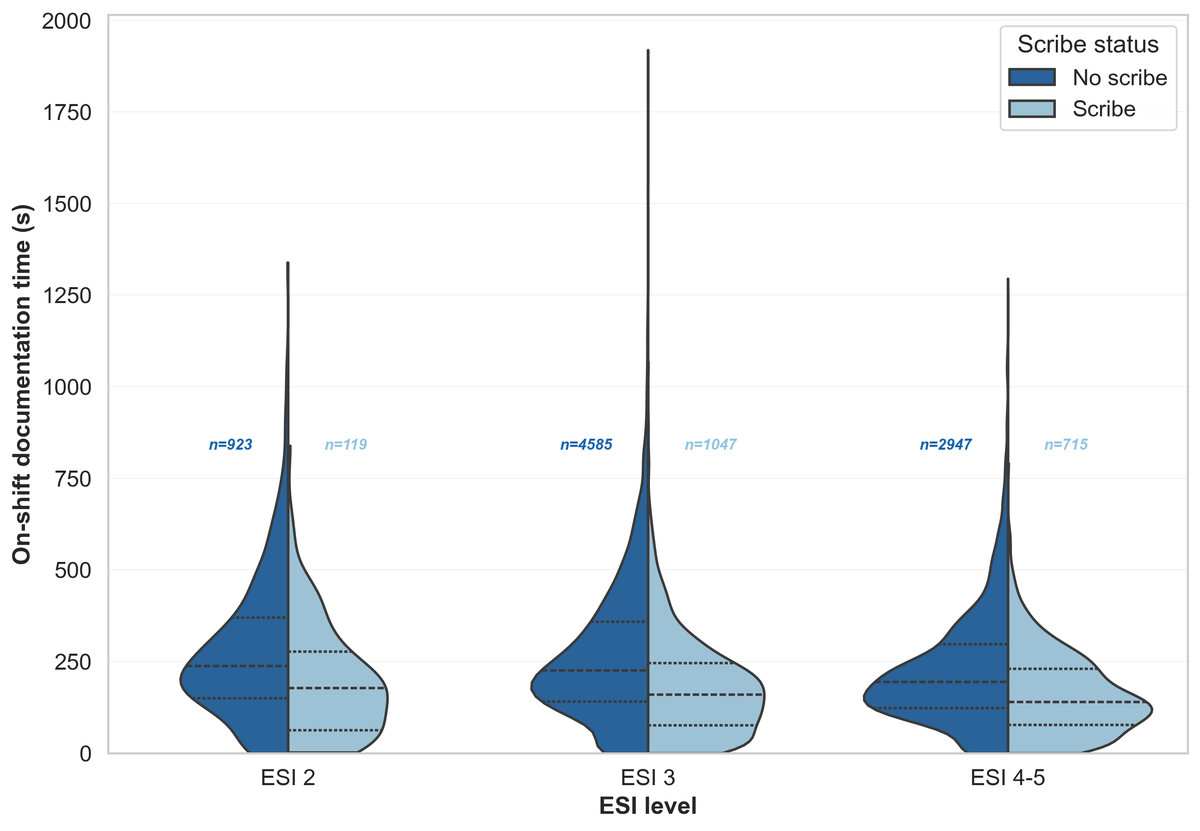
Distribution of on-shift documentation time by acuity level and ambient artificial intelligence (AI) scribe use (n=10,336 encounters; Emergency Severity Index [ESI] level 1 excluded). ESI levels 4 and 5 are combined due to small sample size at ESI level 5 (n=317). Dark blue distributions represent encounters without ambient AI scribes; light blue distributions represent encounters with ambient AI scribes. Dashed lines within each violin plot indicate the median; dotted lines indicate the 25th and 75th percentiles. Wider sections indicate higher density of observations at that documentation time.

In the primary mixed-effects model adjusted for patient and encounter characteristics, ambient AI scribe use was associated with a 72.6-second reduction in on-shift documentation time per encounter (95% CI 63.8-81.4; *P*<.001). Raw mean on-shift documentation time was 173 (SD 131) seconds for ambient scribe encounters vs 254 (SD 183) seconds for non–ambient scribe encounters. The adjusted estimate represents a 28% relative reduction from the model-predicted baseline for a nonscribe encounter with an ESI level 4 patient in a main ED bed. For encounters in which the scribe was used, this corresponds to approximately 24 minutes of documentation time saved over an 8-hour shift if the tool were used across all 20 encounters. The intraclass correlation coefficient for physicians was 0.39. Zone-by-acuity interaction terms collectively improved model fit (likelihood ratio: *c*^2^_9_=86.6; *P*<.001), although most individual interaction terms were not statistically significant (individual *P* values reported in Multimedia Appendix 1). Figure 1 shows the distribution of on-shift documentation time by acuity level and ambient AI scribe use.

### Secondary Outcomes

The results for all secondary outcomes are shown in Table 2. Ambient AI scribe use was associated with a significant 56.6-second reduction in total EHR time per encounter (95% CI 39.8-73.4; *P*<.001; raw mean 529, SD 303 seconds for ambient scribe vs 619, SD 371 seconds for non–ambient scribe) and a 690-character reduction in note length (95% CI 273-1107; *P*=.001; raw mean 9442, SD 5048 characters vs 11,087, SD 8608 characters). After-shift documentation time increased modestly by 9.1 seconds per encounter (95% CI 2.9-15.3; *P*=.004). Both total EHR time and note length increased significantly with higher patient acuity (ESI levels 2 and 3 vs ESI level 4; *P*<.001 in all cases) and greater clinical complexity (number of diagnoses; *P*<.001). All non–main ED clinical zones were associated with significantly shorter notes (reductions of 1727 to 3502 characters; *P*<.001 in all cases).

**Table 2:** Mixed-effects models of ambient artificial intelligence (AI) scribe use on primary and secondary outcomes. All models include random intercepts for clinician (n=100).

Fixed effects		On-shift documentation time^a^ estimate (s; 95% CI)	Total EHR^b^ time^a^ estimate (s; 95% CI)	Note character^c^ estimate (95% CI)
Ambient AI scribe use		−72.6 (−81.4 to −63.8)^d^	−56.6 (−73.4 to −39.8)^d^	−690 (−1107 to −273)^e^
Age (per year)		0.3 (0.2 to 0.5)^d^	1.7 (1.5 to 2.0)^d^	67.9 (60.9 to 74.8)^d^
**Acuity (reference: ESI^f^ level 4)**				
	ESI level 2	48.9 (29.7 to 68.1)^d^	155 (134 to 175)^d^	4451 (3933 to 4969)^d^
	ESI level 3	43.2 (25.8 to 60.7)^d^	84.3 (71.7 to 96.9)^d^	1988 (1674 to 2302)^d^
	ESI level 4	Reference	Reference	Reference
	ESI level 5	−34.8 (−94.2 to 24.5)	−48.3 (−79.1 to −17.5)^e^	−1133 (−1901 to −365)^e^
Number of diagnoses		25.6 (21.5 to 29.8)^d^	87.5 (79.5 to 95.4)^d^	1318 (1121 to 1516)^d^
**Zone group (reference: main ED^g^)**				
	Telemedicine	57.1 (37.7 to 76.5)^d^	43.2 (21.3 to 65.1)^d^	−2562 (−3108 to −2017)^d^
	Triage and lobby	−19.2 (−36.9 to −1.6)^h^	−120 (−136 to −104)^d^	−3502 (−3902 to −3103)^d^
	Vertical care	10.8 (−8.9 to 30.6)	−15.5 (−31.3 to 0.4)	−1727 (−2121 to −1333)^d^

^a^Models include zone × acuity interaction terms (collectively significant; likelihood ratio: *χ*^2^_9_=86.6; *P*<.001; complete results can be found in Multimedia Appendix 1); the coefficients shown are main effects. Sample sizes: n=10,336 for on-shift documentation time (Emergency Severity Index levels 2-5 only) and n=10,344 for total electronic health record time (all Emergency Severity Index levels). Model intercepts and residual variance: intercept=259.5 seconds and σ^2^=19,435 for on-shift documentation time and intercept=655.0 seconds and σ^2^=70,721 for total electronic health record time.

^b^EHR: electronic health record.

^c^Total physician-generated note characters. Sample size: n=10,344. Model intercept=11,871; σ²=4.39 × 10^7^.

^d^*P*<.001.

^e^*P*<.01.

^f^ESI: Emergency Severity Index.

^g^ED: emergency department.

^h^*P*<.05.

### Heterogeneity of Effect on Documentation Time

**Figure 2:**
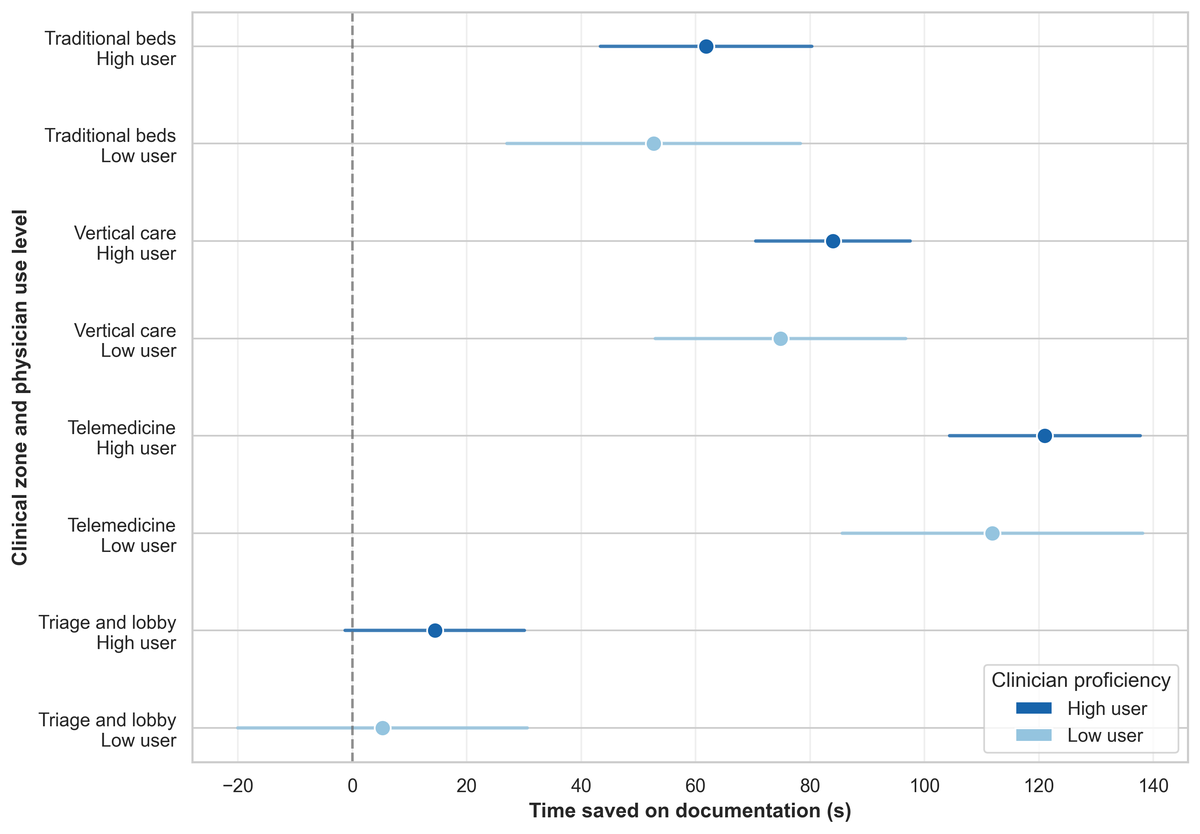
Heterogeneity of ambient scribe effect on on-shift documentation time: model-predicted time savings (seconds) stratified by clinical workflow zone and physician use level. Points represent point estimates; horizontal lines represent 95% CIs. High- and low-use physicians (defined relative to the cohort mean use rate of 18.2%) showed similar per-encounter savings within each zone, with overlapping CIs consistent with the absence of a statistically significant interaction (*P*=.51). The effect varied substantially across clinical zones, with the largest savings in telemedicine and the smallest saving in triage and lobby. Predictions are from mixed-effects models adjusted for patient age, acuity (Emergency Severity Index), and number of diagnoses, with random intercepts for individual physicians. Telemedicine: on-site patients managed via video by a telemedicine emergency department (ED) physician; traditional beds (main ED): stretcher-based care; triage and lobby: rapid assessment area; vertical care: chair-based ambulatory care.

We tested for heterogeneity by physician use level and clinical zone (Figure 2). High-use physicians (use rates of ≥18.2%, which was the cohort mean) had a 71.6-second adjusted reduction per encounter (95% CI 62.8-80.5) compared with 64.2 seconds for low or moderate users; this difference was not statistically significant (*P*=.51).

The effect also varied by clinical zone group. Compared with the 69.4-second time savings in main ED beds, the adjusted benefit was significantly larger in telemedicine (109.9-second reduction; difference of +57.1 seconds; *P*<.001). Vertical care savings (83.6-second reduction) were larger in magnitude than main ED savings (difference of −14.2 seconds; *P*=.20).

### Robustness Analyses

The primary model explained 42.6% of the variance (conditional *R*^2^=0.426). Results were robust across sensitivity analyses, including log transformation, temporal trend adjustments, and doubly robust estimation. Negative control outcomes were largely null or directionally inconsistent with confounding. The within-clinician placebo permutation test yielded a null distribution centered near 0 (mean −0.8 seconds). Complete results are reported in Multimedia Appendix 1.

## Discussion

### Principal Findings

In this single-center analysis spanning 13 months and 10,344 encounters, ambient AI scribe use was associated with a 73-second adjusted reduction in per-encounter on-shift documentation time among emergency physicians, representing a 28% decrease from the model-predicted baseline (260 to 187 seconds). To our knowledge, this is the first inferential evaluation of ambient AI scribes on documentation time in the ED using EHR audit log data, and it extends prior descriptive work with adjusted modeling and a larger sample.

### Comparison With Prior Work

This study extends our prior descriptive analysis of the first 8 months of ED ambient AI scribe rollout at the same institution [16]. This earlier work characterized adoption patterns and reported an unadjusted median documentation time difference of approximately 65 seconds in favor of ambient scribe encounters but did not adjust for patient acuity, complexity, zone, or physician-level clustering. This analysis covered a longer observation window during which adoption matured to 18.2% (1881/10,344) of encounters across 100 contributing physicians; used mixed-effects models with random intercepts for clinician; and incorporated negative control, placebo permutation, sensitivity, and doubly robust analyses. The adjusted estimate (−73 seconds) is closely aligned with the unadjusted descriptive median (−65 seconds), suggesting that the observed time savings are not an artifact of early adopter case selection and are likely to persist as adoption broadens.

Our 28% reduction in documentation time aligns with the magnitude of effect reported in other ambient AI scribe studies, although important differences emerged in secondary outcomes. The Permanente Medical Group’s 63-week evaluation across multiple specialties reported significant reductions in note-taking time and after-hours documentation (“pajama time”) [8], and a primary care study found a 30% decrease in after-hours EHR time [12]. In contrast, we observed only a small increase in after-shift documentation time (approximately 9 seconds per encounter). This divergence likely reflects differences between ambulatory and ED workflows. In the ED, notes are often completed in near real time, meaning that on-shift efficiency gains may not translate to measurable reductions in discrete after-shift activities. The small increase in after-shift time may represent physicians choosing to perform final edits and reviews of AI-generated notes outside their clinical shifts, effectively shifting rather than eliminating some documentation burden.

We also observed a 690-character reduction in note length, contrasting with some ambulatory care studies reporting increased note length under ambient AI scribe use [14]. This may reflect reduced reliance on templated text or removal of extraneous content, although further study of note quality is needed to determine whether brevity reflects efficiency or loss of clinical detail.

Most prior ambient AI scribe research has evaluated scheduled, ambulatory encounters in primary care, mental health, and specialty clinics with predictable workflows and dedicated appointment times [7,11,13]. The ED environment, which includes unscheduled visits, frequent interruptions, variable patient complexity, time pressure, simultaneous patient management, and high cognitive load, represents a distinct challenge for documentation technology. While human medical scribes have demonstrated improved ED throughput and productivity in systematic reviews [20], AI scribes offer advantages in scalability and ubiquitous availability. Our findings extend the ambient AI scribe literature to the ED setting, suggesting that the technology can function effectively even in demanding, unpredictable clinical conditions.

### Clinical Implications

The reduction of 73 seconds per encounter that we observed translates to approximately 24 minutes of documentation time saved over a typical 8-hour shift if the tool were used across 20 encounters; for any individual physician, the per-shift savings scales with the proportion of encounters on which they choose to use the tool. This time could be reoriented toward direct patient care, decision-making, communication, or trainee supervision. Given that documentation burden is a major contributor to physician burnout, even modest per-encounter savings may contribute to meaningful effects on physician well-being [1,4]. The 57-second reduction in total EHR time per encounter was smaller than the on-shift documentation savings, suggesting that some burden may have shifted to other periods or activities.

The substantial physician-level variability we observed (39% of the variance in documentation time was attributable to individual physician practice patterns) has direct implementation consequences. Notably, however, high- and low- or moderate-use physicians realized comparable per-encounter savings (−71.6 vs −64.2 seconds; interaction *P*=.51), in contrast to some prior implementations where heavier users have shown disproportionately greater benefits [8]. This finding suggests that the per-encounter benefit is largely conferred whenever the tool is engaged rather than accruing only for physicians who have integrated it into most of their visits and that policies oriented toward broad availability may capture meaningful gains even without uniformly high adoption. Individual documentation styles, patient case mix, baseline documentation burden, and comfort with editing AI-generated content likely still drive who chooses to adopt, but the magnitude of benefit per ambient scribe–supported encounter does not appear contingent on use frequency.

The zone-level heterogeneity we observed (significantly larger effects in telemedicine, *P*<.001 and a significantly smaller effect in the triage and lobby zone, *P*<.001 compared with main ED beds, with vertical care being similar to the main ED) further underscores that workflow context, more than physician-level use frequency, influences the per-encounter efficiency gain. This pattern suggests that ambient AI scribes are best suited for workflows with linear, lower-interruption visit structures (most strikingly telemedicine) and may underperform in rapid assessment areas where short patient interactions and high task switching limit the time available for the tool to add value. From an implementation perspective, this argues for zone-targeted rollout strategies and tempered expectations in workflows that diverge from the ambulatory-style use cases for which these tools were originally designed.

### Limitations

Several limitations warrant consideration. First, this was a single-center study at a tertiary academic ED; generalizability to community hospitals, smaller academic centers, or non-Epic environments remains untested. Second, ambient AI use was voluntary and entirely at attending physician discretion. Although we adjusted for patient acuity, complexity, zone, and physician-level effects, and although we observed that associations were robust across multiple sensitivity, falsification, and doubly robust analyses, we cannot fully exclude unmeasured confounding. In particular, even within strata of measured covariates, physicians may have selectively used ambient AI for encounters they anticipated would be straightforward in ways that our covariates did not capture (eg, anticipated patient cooperativeness, complexity of the social or psychiatric history, or expected documentation length). Our negative control outcomes (showing null or trivial effects on nondocumentation EHR activities) and within-clinician placebo permutation test argue against this pattern but do not eliminate it. Accordingly, we frame our findings as adjusted associations rather than causal effects.

Third, outcomes were derived from EHR audit logs, which estimate active EHR time using a 5-second inactivity threshold but cannot fully distinguish focused documentation from concurrent multitasking, brief interruptions, or background clinical activity. Misclassification of on-shift vs after-shift work is possible when physicians extend beyond scheduled shift end or document remotely. Fourth, while overall adoption reached 18.2% (1881/10,344) over the 13-month observation window, this still represents an early rollout phase, and steady-state performance after broader, more uniform adoption may differ; the reported effect should not be extrapolated to encounters or workflows outside the observed distribution. Fifth, we did not directly assess documentation quality, coding accuracy, billing impact, patient experience, or financial outcomes; all are critical dimensions of a comprehensive evaluation that remain for future work.

### Future Directions

Several lines of inquiry follow directly from this work. Multicenter studies are needed to establish generalizability across institutional contexts and EHR configurations. Prospective designs with concurrent controls (including stepped-wedge or cluster randomized rollouts) would substantially strengthen causal inference beyond what observational analyses can provide. Evaluations of note quality, downstream coding and billing, and patient experience outcomes are essential before broad adoption can be unambiguously recommended. Critically, return on investment analyses incorporating licensing costs; training time; integration overhead; and downstream effects on throughput, quality, and clinician retention are needed to determine whether the per-encounter time savings observed in this study justify the financial and operational costs of large-scale deployment. Finally, identifying the physician, patient, and workflow characteristics that predict the greatest benefits would enable targeted implementation strategies that maximize value while concentrating resources where they are likely to matter most.

### Conclusions

In this single-center analysis of a 13-month staged rollout, ambient AI scribe use was associated with a 28% adjusted reduction in per-encounter on-shift documentation time among emergency physicians. Effects on secondary outcomes (a small increase in after-shift documentation time and a modest decrease in note length) differ from those of some ambulatory care studies, likely reflecting ED-specific workflow patterns. The per-encounter benefit appeared consistent across physicians irrespective of use frequency but varied modestly by clinical workflow zone, suggesting that effectiveness depends more on contextual workflow factors than on individual adoption intensity. This work contributes the first objective, audit log–derived inferential evidence on ambient AI scribe effects in the ED, extending prior descriptive findings and providing a benchmark against which future multicenter, prospective, and quality-focused evaluations can be calibrated.

## Appendix

Multimedia Appendix 1 Extended statistical methods, robustness and sensitivity analyses, supplementary tables, additional model diagnostics, zone × acuity interaction analysis, patient demographics and sensitivity analysis, and software and computation.

## Abbreviations

AI: artificial intelligence
ED: emergency department
EHR: electronic health record
ESI: Emergency Severity Index
STROBE: Strengthening the Reporting of Observational Studies in Epidemiology

## Data Availability

The datasets generated or analyzed during this study are available from the corresponding author on reasonable request.
