# Supplementary material for: Ambient AI Scribes and Emergency Department Documentation Burden: Retrospective Cohort Study
Source: JMIR AI. 2026 Jul 2;5:e92193. doi: 10.2196/92193 (PMC13376842; doi:10.2196/92193)
Supplement: Multimedia Appendix 1 [file ai_v5i1e92193_app1.docx]

**Multimedia Appendix 1: Supplementary Methods and Results**

*Effect of Ambient Artificial Intelligence Scribes on Documentation Burden in the Emergency Department*

## Table of Contents

1. Extended Statistical Methods

2. Robustness and Sensitivity Analyses

3. Supplementary Tables

4. Additional Model Diagnostics

5. Zone × Acuity Interaction Analysis

6. Patient Demographics and Sensitivity Analysis

7. Software and Computation

# 1. Extended Statistical Methods

## 1.1 Data Sources and Processing

**Electronic Health Record Data Extraction**

Data were extracted from the Epic EHR system (Epic Systems Corporation, Verona, WI) using two primary data sources:

1. **Active Use Log (UAL Lite):** Epic's UAL Lite provides developer-derived measures of user activity duration. This system tracks user interactions with specific EHR activities and aggregates them at the hourly level. Time measurements use Epic's standard methodology with a five-second timeout threshold for inactivity. Individual user interactions within each hour are aggregated by Epic's proprietary algorithms to produce total active time per activity category.

2. **Note Attribution Table:** Contains clinical documentation metadata including: total character counts by author type (attending, resident, scribe); ambient AI-generated character counts; entry method timestamps; note completion timestamps; and attribution metadata.

**Activity Classification**

"Notes" activities were defined using an established categorization schema developed by Epic analysts and validated in prior health services research. Raw Epic activity codes were mapped to clinically meaningful documentation tasks through a validated crosswalk table. This mapping has been used in prior published studies of physician EHR time.

**Temporal Boundaries**

Attending physician shift schedules were obtained from the clinical scheduling system (Lightning Bolt Solutions). Activity times were aggregated at the patient encounter level and classified as either on-shift or after-shift based on the following rules: on-shift activities occurred between shift start and shift end; after-shift activities occurred >15 minutes after shift end (15-minute buffer to account for typical sign-out procedures).

**Encounter-Level Normalization**

All time metrics were normalized to the individual patient encounter level. For encounters spanning multiple shifts or involving multiple providers, only time attributable to the primary attending physician was included.

## 1.2 Ambient Scribe Identification

Encounters utilizing ambient AI scribes were identified through the presence of nonzero values in the total_attending_chars_ambient field in the note attribution table. This field is automatically populated by Epic when the DAX Copilot system submits generated text for attending physician review.

**Validation:** We validated this identification method through manual chart review of 100 randomly selected encounters (50 ambient, 50 nonambient) with 100% agreement between automated classification and manual review.

## 1.3 Covariate Selection and Specification

**Age:** Patient age at ED arrival, mean-centered to facilitate interpretation of the intercept.

**Acuity:** Emergency Severity Index (ESI) level, a validated 5-level triage system where 1 = highest acuity and 5 = lowest acuity. ESI level 4 was used as the reference category. ESI 1 encounters (n = 8) were excluded from the primary zone × acuity model because the small cell sizes precluded stable estimation of the interaction terms involving ESI 1; a sensitivity analysis with ESI 1 included yielded an effectively identical primary effect.

**Number of ED Diagnoses:** Count of ICD-10 diagnosis codes assigned to the encounter, mean-centered.

**Clinical Zone:** Four distinct ED workflow zones: **Main ED (Traditional Beds):** stretcher-based rooms for moderate-acuity patients not requiring resuscitation-level care; **Vertical Care:** chair-based care areas for ambulatory patients with lower-acuity conditions; **Triage and Lobby:** rapid assessment and treatment area for patients who can be evaluated and discharged quickly; **Telemedicine:** on-site patients evaluated via video by ED physicians in a remote telemedicine command center.

**Exclusions:** Walk-in Clinic encounters were excluded because they do not undergo formal ESI triage; resuscitation and trauma encounters were excluded due to distinct workflows and documentation practices; pediatric ED encounters were excluded due to age-specific workflows.

## 1.4 Statistical Model Specifications

**Primary Model Formula:**

notes_sec_on_shift ~ ambient_exposure +

age_at_arrival_years_centered +

num_ed_dx_enc_centered +

C(acuity_imputed, Treatment(reference=4)) +

C(zone_group, Treatment(reference='Traditional Beds')) +

C(acuity_imputed) : C(zone_group) +

(1 | clinician_user_id)

**Interpretation of Coefficients:** ambient_exposure is the mean difference in seconds between ambient and nonambient encounters, adjusted for all covariates. Zone and acuity main effects represent differences relative to reference categories. Zone × acuity interaction terms represent additional effects for specific zone-acuity combinations beyond additive effects.

**Random Effects Structure:** Random intercepts for clinician allow each physician to have their own baseline documentation time, accounting for individual practice patterns and documentation styles.

## 1.5 Model Diagnostics

**Multicollinearity Assessment**

Variance Inflation Factors (VIF) were calculated for all predictors in the main-effects specification:

| **Variable** | **VIF** | **Tolerance** |
| --- | --- | --- |
| ESI 3 (vs ESI 4) | 1.89 | 0.53 |
| Vertical Care (vs Traditional Beds) | 1.67 | 0.60 |
| Ambient exposure | 1.32 | 0.76 |
| Telemedicine (vs Traditional Beds) | 1.31 | 0.76 |
| Triage and Lobby (vs Traditional Beds) | 1.30 | 0.77 |
| Age (centered) | 1.18 | 0.85 |
| ESI 2 (vs ESI 4) | 1.09 | 0.92 |
| ESI 5 (vs ESI 4) | 1.07 | 0.94 |
| Number of diagnoses (centered) | 1.01 | 0.99 |

All VIF values <2.5 indicate no problematic multicollinearity. The condition number of the design matrix is 120.

**Residual Diagnostics**

Q-Q plots showed approximate normality of residuals with some deviation in the tails, consistent with the right-skewed nature of time data; sensitivity analyses using log-transformed and Gamma-link models (Section 2) accommodate this skew explicitly. Residuals grouped by physician showed no systematic patterns, confirming the random-effects structure adequately accounts for within-physician clustering.

**Justification for Linear Model with Right-Skewed Outcomes**

Despite right-skewed outcome distributions (skewness 1.51–4.54; Section 4.1), linear mixed-effects models are appropriate because: (1) the large sample size (n=10,344) ensures the Central Limit Theorem applies to estimation of mean differences; (2) medians [IQR] are reported for descriptive statistics while the LMM is used for inference on mean differences; (3) sensitivity analyses with log-transformed outcomes (Section 2.1) and Gamma GEE models (Section 2.2) yield consistent results; (4) coefficients in original units (seconds) are more interpretable for clinical audiences; and (5) this approach is standard in health services research for time-based outcomes.

# 2. Robustness and Sensitivity Analyses

## 2.1 Log-Transformation Sensitivity Analysis

**Rationale:** Log transformation of skewed outcomes can normalize distributions and stabilize variance.

**Method:** Outcome was log(notes_sec_on_shift + 1); same model structure as primary analysis. Exponentiated coefficients represent proportional changes.

**Results:** Ambient log coefficient: −0.4784 (95% CI [−0.5424, −0.4127], P<.001). Proportional change: −38.0% (95% CI [−42.0%, −33.8%]). Translated to absolute scale at the nonambient mean (253.6s), this corresponds to approximately −96.4s.

**Conclusion:** Consistent in direction and significance with the primary analysis (−72.6s).

## 2.2 Gamma Generalized Estimating Equations

**Rationale:** The Gamma distribution is appropriate for positive, right-skewed continuous outcomes.

**Method:** Gamma family with log link, GEE with clinician clustering; same covariate structure as primary model.

**Results:** Ambient log coefficient: −0.4298 (95% CI [−0.5985, −0.2604], P<.001). Proportional change: −34.9% (95% CI [−45.1%, −22.9%]).

**Conclusion:** Consistent in direction and significance with the primary analysis.

## 2.3 Temporal Trend Adjustments

### 2.3.1 Week Fixed Effects

**Method:** Added fixed effects for each calendar week across the full study period.

**Results:** Ambient coefficient: −68.1s (95% CI [−77.3, −59.0], P<.001).

**Conclusion:** Effect mildly attenuated but remains highly significant.

### 2.3.2 Month Fixed Effects

**Method:** Added fixed effects for each calendar month.

**Results:** Ambient coefficient: −68.9s (95% CI [−77.9, −59.8], P<.001).

**Conclusion:** Consistent with the primary analysis.

### 2.3.3 Linear Time Trend

**Method:** Added a continuous time variable (weeks since study start).

**Results:** Ambient coefficient: −68.9s (95% CI [−77.9, −59.9], P<.001). Time trend: −0.33s per week (95% CI [−0.50, −0.15], P<.001).

**Conclusion:** A modest secular decline in on-shift documentation time was detected; the ambient effect is robust to this adjustment.

### 2.3.4 Quadratic Time Trend

**Method:** Added linear and quadratic week-since-start terms.

**Results:** Ambient coefficient: −68.3s (95% CI [−77.3, −59.3], P<.001). Linear term: −0.81s/week (P=.018); quadratic term: +0.0088 (P=.14).

**Conclusion:** No statistically significant nonlinear time trend; the ambient effect is unchanged after additional flexibility in the time term.

## 2.4 Doubly Robust Estimation (Inverse Probability Weighting)

**Step 1 — Propensity Score Model.** Logistic regression predicting ambient scribe use, with age, number of diagnoses, ESI, and zone as covariates.

**Step 2 — Stabilized Weight Calculation.** Stabilized weights SW = P(A=a) / P(A=a|X), capped at the 99th percentile (1.90) to limit influence of extreme weights.

**Step 3 — Weighted Estimation.** A weighted least-squares model on the on-shift documentation outcome with cluster-robust standard errors at the clinician level produced the marginal IPW estimate. A doubly robust estimate was obtained from a weighted regression that additionally adjusted for the same covariates used in the propensity-score model.

**Results:** IPW (marginal ATE, clinician-clustered SE): −83.8s (95% CI [−118.1, −49.5], P<.001). Doubly robust (weighted + adjusted): −83.2s (95% CI [−116.6, −49.8], P<.001).

**Conclusion:** Doubly robust estimates remain large and highly significant. Both are slightly larger in magnitude than the primary mixed-effects estimate (−72.6s), suggesting that any residual confounding is more likely to attenuate than inflate the primary effect.

**Weight Diagnostics.** Mean stabilized weight: 1.00; SD: 0.27; range: [0.43, 1.90]; 1st–99th percentile: [0.44, 1.90]. Mean propensity score, treated: 0.245; controls: 0.168. Effective sample size: 9,616 (93.0% of the 10,336 weighted observations).

## 2.5 Placebo Exposure Test

**Method:** Each encounter's true ambient exposure was randomly permuted within clinician (preserving each physician's overall ambient rate but breaking the encounter-to-exposure link). The primary model was refit to this placebo exposure on 200 independent permutations to generate an empirical null distribution.

**Results:** Observed ambient coefficient: −72.4s. Placebo distribution: mean −0.78s, SD 3.87s, range [−12.8s, +9.0s]. Empirical 2-sided P-value (|placebo| ≥ |observed|): 0/200 (P<.005).

**Conclusion:** The observed effect is far outside the empirical null distribution, supporting a treatment-related rather than artifactual interpretation.

## 2.6 Dose-Response Analysis

### 2.6.1 Continuous Ambient Proportion

**Method:** Re-fit the primary model replacing the binary ambient indicator with a continuous exposure equal to the proportion of attending note characters generated by the ambient scribe.

**Results:** Coefficient: −202.9s per 100% increase (95% CI [−224.6, −181.2], P<.001). Each 10-percentage-point increase in ambient proportion corresponds to a −20.3s reduction in on-shift documentation time.

**Conclusion:** A monotonic dose-response gradient is observed.

### 2.6.2 Threshold Analysis

**Method:** Tested a threshold at 10% ambient proportion (≥10% vs <10% ambient content).

**Results:** Coefficient (≥10% vs <10%): −72.8s (95% CI [−81.7, −64.0], P<.001) — very similar to the binary ambient vs nonambient comparison (−72.6s).

**Conclusion:** The effect is present across the exposure range and is not driven by encounters with only minimal ambient content.

## 2.7 Negative Control Outcomes (Falsification Tests)

**Rationale:** Test the specificity of the ambient scribe effect on documentation activities. Ambient AI scribes generate text in clinical notes; they do not directly produce orders, in-basket messages, or chart-review activity, so coefficients on these outcomes should be small or null.

**Method:** The primary mixed-effects specification was applied to four control outcomes alongside the on-shift documentation outcome (positive control).

| **Outcome** | **n** | **Coefficient (s)** | **95% CI** | **P-value** |
| --- | --- | --- | --- | --- |
| **On-shift documentation (positive control)** | 10,336 | **−72.4** | **[−81.2, −63.6]** | **<.001** |
| On-shift order entry | 10,336 | +4.0 | [−1.3, +9.4] | .14 |
| On-shift in-basket | 10,336 | +1.11 | [+0.49, +1.74] | <.001 |
| After-shift in-basket | 10,336 | −0.001 | [−0.19, +0.18] | .99 |
| After-shift chart review | 10,336 | +0.07 | [−1.80, +1.94] | .94 |

**Interpretation:** The two after-shift activities (in-basket and chart review) show effectively null coefficients (|coef| ≤ 0.1s, P ≥ .94), on-shift order entry is null at the conventional threshold (+4.0s, P=.14), and on-shift in-basket shows a small positive coefficient (+1.1s, P<.001) — an order of magnitude smaller than the primary documentation effect (−72.6s) and in the opposite direction. The pattern is mechanistically consistent with reallocation of a small portion of freed-up shift time toward in-basket review rather than a generic measurement artifact. The positive-control re-fit recovers the primary effect to within 0.04s, confirming the negative-control pipeline is correctly specified.

## 2.8 Outlier Robustness

### 2.8.1 Winsorization

**Method:** The on-shift documentation outcome was winsorized at the 1st and 99th percentiles (clip range 0.0–820.6s) and the primary model was refit.

**Results:** Ambient coefficient: −70.8s (95% CI [−79.2, −62.4], P<.001).

**Conclusion:** Robust to extreme values.

### 2.8.2 Trimming

**Method:** Encounters with on-shift documentation time above the 95th percentile (>580s, n=517 dropped, n=9,819 retained) were excluded.

**Results:** Ambient coefficient: −57.5s (95% CI [−64.7, −50.3], P<.001).

**Conclusion:** The effect is attenuated as expected when the right tail is removed but remains highly significant, confirming the result is not driven solely by a small number of extreme outliers.

# 3. Supplementary Tables

## Table S1. Complete Model Coefficients for Primary Analysis

| **Fixed Effect** | **Coefficient (seconds)** | **95% CI** | **P-value** |
| --- | --- | --- | --- |
| **Intercept** | 259.5 | [232.1, 286.9] | <.001 |
| **Ambient Exposure** | **−72.6** | **[−81.4, −63.8]** | **<.001** |
| Age (per year) | +0.33 | [+0.18, +0.47] | <.001 |
| Number of ED Diagnoses | +25.6 | [+21.5, +29.8] | <.001 |
| **ESI (ref: ESI 4)** |  |  |  |
| ESI 2 | +48.9 | [+29.7, +68.1] | <.001 |
| ESI 3 | +43.2 | [+25.8, +60.7] | <.001 |
| ESI 5 | −34.8 | [−94.2, +24.5] | .25 |
| **Zone (ref: Main ED or Traditional Beds)** |  |  |  |
| Vertical Care | +10.8 | [−8.9, +30.6] | .28 |
| Triage and Lobby | −19.2 | [−36.9, −1.6] | .033 |
| Telemedicine | +57.1 | [+37.7, +76.5] | <.001 |
| **Zone × Acuity Interactions** |  |  |  |
| ESI 2 × Vertical Care | −9.4 | [−45.1, +26.3] | .61 |
| ESI 2 × Triage and Lobby | −18.1 | [−45.6, +9.5] | .20 |
| ESI 2 × Telemedicine | −66.4 | [−172.1, +39.4] | .22 |
| ESI 3 × Vertical Care | −11.3 | [−32.8, +10.3] | .31 |
| ESI 3 × Triage and Lobby | −31.7 | [−51.8, −11.5] | .002 |
| ESI 3 × Telemedicine | +0.1 | [−23.4, +23.6] | .99 |
| ESI 5 × Vertical Care | +28.6 | [−47.9, +105.1] | .46 |
| ESI 5 × Triage and Lobby | +6.6 | [−57.0, +70.2] | .84 |
| ESI 5 × Telemedicine | +24.3 | [−41.7, +90.2] | .47 |

**Random Effects:** Physician variance: 12,409 s²; Residual variance: 19,435 s²; Physician ICC: 0.390.

**Model Fit:** Conditional R²: 0.426 (42.6% variance explained); Marginal R²: 0.060 (6.0% by fixed effects alone); Likelihood-ratio test for zone × acuity interactions: χ² = 86.6, df = 9, P<.001.

## Table S2. Summary of All Robustness and Sensitivity Analyses

| **Analysis** | **Ambient effect** | **95% CI** | **vs Primary** |
| --- | --- | --- | --- |
| **Primary mixed-effects model** | **−72.6s** | **[−81.4, −63.8]** | reference |
| Log-LMM (% change) | −38.0% (≈−96.4s) | [−42.0%, −33.8%] | larger* |
| Gamma GEE (% change) | −34.9% | [−45.1%, −22.9%] | larger* |
| Linear time trend | −68.9s | [−77.9, −59.9] | −5.1% smaller |
| Week fixed effects | −68.1s | [−77.3, −59.0] | −6.2% smaller |
| Month fixed effects | −68.9s | [−77.9, −59.8] | −5.1% smaller |
| Quadratic time trend | −68.3s | [−77.3, −59.3] | −5.9% smaller |
| Winsorized 1%–99% | −70.8s | [−79.2, −62.4] | −2.5% smaller |
| Trimmed >P95 | −57.5s | [−64.7, −50.3] | −20.8% smaller |
| Inverse probability weighting (ATE) | −83.8s | [−118.1, −49.5] | +15.4% larger |
| Doubly robust (IPW + adjustment) | −83.2s | [−116.6, −49.8] | +14.6% larger |
| Demographics-adjusted | −71.7s | [−80.5, −62.9] | −1.3% smaller |
| Dose-response, threshold ≥10% ambient | −72.8s | [−81.7, −64.0] | +0.3% larger |
| ESI-1 included (main effects only) | −72.4s | [−81.2, −63.6] | −0.2% smaller |

*Log-link models report proportional change; absolute-second translation uses the nonambient mean (253.6s).*

**Conclusion:** The primary estimate is robust across all sensitivity analyses. The two methodological extremes — log-link models that down-weight long tails, and IPW and doubly robust models that up-weight under-represented ambient encounters — bracket the primary estimate, with neither direction crossing the null. Trimming at the 95th percentile attenuates the effect by ~22% but it remains highly significant, confirming the result is not driven solely by long-tail observations.

## Table S3. Negative Control Outcomes – Detailed Results

| **Outcome** | **n** | **Coefficient** | **95% CI** | **P-value** |
| --- | --- | --- | --- | --- |
| **On-shift documentation (positive control)** | 10,336 | **−72.4s** | **[−81.2, −63.6]** | **<.001** |
| On-shift order entry | 10,336 | +4.0s | [−1.3, +9.4] | .14 |
| On-shift in-basket | 10,336 | +1.11s | [+0.49, +1.74] | <.001 |
| After-shift in-basket | 10,336 | −0.001s | [−0.19, +0.18] | .99 |
| After-shift chart review | 10,336 | +0.07s | [−1.80, +1.94] | .94 |

The two after-shift activities show effectively null effects, on-shift order entry is null at the conventional threshold, and on-shift in-basket shows a small positive coefficient in the *opposite* direction of the primary effect, an order of magnitude smaller in absolute size. This is consistent with reallocation of a small portion of freed-up shift time toward in-basket review rather than a generic measurement artifact, and it supports specificity of the ambient scribe effect on documentation activities. The positive-control re-fit recovers the primary effect within 0.04s, confirming the negative-control pipeline is correctly specified.

## Table S4. Model Comparison (Nested Specifications)

| **Model** | **Fixed-effect parameters** | **Conditional R²** | **Marginal R²** | **ICC** | **Log-likelihood** |
| --- | --- | --- | --- | --- | --- |
| Primary (with zone × acuity) | 19 | 0.426 | 0.060 | 0.390 | −65,825.5 |
| Main effects only | 10 | 0.426 | 0.059 | 0.390 | −65,868.8 |
| Ambient only (unadjusted) | 2 | 0.396 | 0.012 | 0.389 | −66,230.1 |

The likelihood-ratio test comparing the model with vs without zone × acuity interactions yields χ² = 86.6, df = 9, P<.001, supporting inclusion of the interaction terms.

# 4. Additional Model Diagnostics

## 4.1 Distribution of Outcomes

| **Outcome** | **Skewness** | **Excess kurtosis** | **Median** | **Mean (SD)** |
| --- | --- | --- | --- | --- |
| On-shift documentation time | 1.51 | 4.25 | 204s | 239.0 (177.3) |
| After-shift documentation time | 4.54 | 31.47 | 0s | 41.2 (111.6) |
| Total EHR time | 1.66 | 4.85 | 516s | 602.3 (361.3) |
| Note characters | 3.42 | 24.57 | 8,808 | 10,788 (8,103) |

All outcomes show right-skewed distributions, typical for time-based and count-based healthcare metrics. The log-LMM and Gamma-GEE sensitivity analyses (Sections 2.1 and 2.2) explicitly accommodate this skew and yield results consistent with the primary linear model.

## 4.2 Correlation Among Predictors

Pearson correlations among continuous predictors:

|  | **Age** | **# Diagnoses** | **Ambient** |
| --- | --- | --- | --- |
| Age | 1.000 | 0.053 | −0.063 |
| # Diagnoses | 0.053 | 1.000 | −0.014 |
| Ambient exposure | −0.063 | −0.014 | 1.000 |

All pairwise correlations are weak (|r| ≤ 0.07), consistent with the low VIF values reported in Section 1.5.

## 4.3 Missing Data Analysis

Total encounters meeting inclusion criteria: 10,344. Encounters used in the primary zone × acuity model (after dropping ESI 1, n = 8): 10,336. Missing key variables in the analytic dataset: 0 (0.0%).

For 72 encounters (0.7% of the analytic cohort), one or more of the demographic fields (pat_language, interpreter_needed, ethnic_group_name, race, sex) or encounter-level fields (acuity_level_c, means_of_arrival_c, first_chief_complaint_id) were initially null in the primary derived tables. Targeted manual review against the structured patient demographics and encounter tables in the EHR recovered values for these fields, which were then merged into the analytic dataset. Imputed values are drawn exclusively from structured EHR fields and represent the same underlying data source as the primary cohort extract; they were therefore treated as ordinary observed values for analysis. After this overlay, no imputation procedure was applied to any analytic field.

## 4.4 Balance Diagnostics

Standardized mean differences (SMDs) between ambient (n=1,881) and nonambient (n=8,463) groups for the model covariates:

| **Variable** | **Ambient (%)** | **Nonambient (%)** | **SMD** | **Balance** |
| --- | --- | --- | --- | --- |
| Age (continuous) | – | – | 0.17 | Good (<0.25) |
| Number of ED diagnoses (continuous) | – | – | 0.04 | Excellent (<0.10) |
| ESI 1 | 0.00 | 0.09 | 0.04 | Excellent |
| ESI 2 | 6.33 | 10.91 | 0.16 | Good |
| ESI 3 | 55.66 | 54.18 | 0.03 | Excellent |
| ESI 4 | 34.77 | 31.80 | 0.06 | Excellent |
| ESI 5 | 3.24 | 3.02 | 0.01 | Excellent |
| Zone: Traditional Beds | 13.61 | 28.34 | 0.37 | Moderate |
| Zone: Vertical Care | 38.28 | 26.05 | 0.26 | Moderate |
| Zone: Triage and Lobby | 18.08 | 35.46 | 0.40 | Moderate |
| Zone: Telemedicine | 30.04 | 10.15 | 0.51 | Poor |

Continuous variables and the ESI distribution are well-balanced (SMD ≤ 0.20). Zone distributions are imbalanced — ambient encounters are over-represented in Telemedicine and Vertical Care and under-represented in Traditional Beds and Triage and Lobby — reflecting differential adoption of ambient scribes across ED workflows during the study period. This imbalance is the principal motivation for (a) regression adjustment for zone in the primary model and (b) the IPW and doubly robust sensitivity analysis (Section 2.4), which produces an estimate that is similar in direction but slightly larger in magnitude than the primary model.

# 5. Zone × Acuity Interaction Analysis

## 5.1 Overall Test of Interactions

To account for differential baseline documentation patterns across clinical workflows, the primary mixed-effects model included interaction terms between clinical zone and patient acuity (ESI level). A likelihood-ratio test comparing the model with interaction terms to a main-effects-only model showed that the interactions collectively improved model fit (LR χ² = 86.6, df = 9, P<.001), justifying their inclusion.

## Table S5. Zone × Acuity Interaction Terms from Primary Model

| **Interaction Term** | **Coefficient (seconds)** | **95% CI** | **P-value** |
| --- | --- | --- | --- |
| **Telemedicine Zone** |  |  |  |
| ESI 2 × Telemedicine | −66.4 | [−172.1, +39.4] | .22 |
| ESI 3 × Telemedicine | +0.1 | [−23.4, +23.6] | .99 |
| ESI 5 × Telemedicine | +24.3 | [−41.7, +90.2] | .47 |
| **Triage and Lobby Zone** |  |  |  |
| ESI 2 × Triage and Lobby | −18.1 | [−45.6, +9.5] | .20 |
| ESI 3 × Triage and Lobby | −31.7 | [−51.8, −11.5] | .002 |
| ESI 5 × Triage and Lobby | +6.6 | [−57.0, +70.2] | .84 |
| **Vertical Care Zone** |  |  |  |
| ESI 2 × Vertical Care | −9.4 | [−45.1, +26.3] | .61 |
| ESI 3 × Vertical Care | −11.3 | [−32.8, +10.3] | .31 |
| ESI 5 × Vertical Care | +28.6 | [−47.9, +105.1] | .46 |

Reference categories: ESI 4 (acuity) and Main ED or Traditional Beds (zone). Coefficients represent the additional effect of being in a specific zone × acuity combination beyond the additive main effects.

**Interpretation:**

1. **One individually significant interaction.** Only ESI 3 × Triage and Lobby reached α = 0.05 (−31.7s, P=.002), indicating that ESI-3 patients in the Triage and Lobby zone require approximately 32 seconds less on-shift documentation than predicted by the additive main effects. The remaining 8 interaction terms are not individually significant.

2. **Collectively significant block.** The likelihood-ratio test (LR χ² = 86.6, df = 9, P<.001) demonstrates that the interaction block as a whole significantly improves model fit.

3. **Clinical implication.** The interactions primarily capture differential baseline documentation time (i.e., workflow complexity) rather than differential AI scribe effectiveness across subgroups. Heterogeneity of the *ambient* effect itself by zone is summarized in the main manuscript Figure 2 and Table 2 and shows attenuation, not a sign change, across zones.

4. **Model justification.** The significant LR test supports including these interaction terms to properly adjust for ED workflow heterogeneity, yielding an unbiased estimate of the ambient AI scribe effect.

# 6. Patient Demographics and Sensitivity Analysis

## 6.1 Detailed Demographic Characteristics

### Table S6. Complete Patient Demographics by Ambient Scribe Use

| **Characteristic** | **No Ambient Scribe (n=8,463)** | **With Ambient Scribe (n=1,881)** |
| --- | --- | --- |
| **Sex, n (%)** |  |  |
| Female | 4,556 (53.8%) | 1,049 (55.8%) |
| Male | 3,904 (46.1%) | 832 (44.2%) |
| Unknown | 3 (0.0%) | 0 (0.0%) |
| **Race, n (%)** |  |  |
| Other | 3,233 (38.2%) | 799 (42.5%) |
| White | 2,670 (31.5%) | 513 (27.3%) |
| Asian | 1,318 (15.6%) | 286 (15.2%) |
| Black or African American | 497 (5.9%) | 103 (5.5%) |
| Native Hawaiian or Other Pacific Islander | 181 (2.1%) | 57 (3.0%) |
| American Indian or Alaska Native | 35 (0.4%) | 4 (0.2%) |
| Unknown or Declined | 529 (6.3%) | 119 (6.3%) |
| **Ethnicity, n (%)** |  |  |
| Non-Hispanic or Non-Latino | 5,057 (59.8%) | 1,051 (55.9%) |
| Hispanic or Latino | 3,137 (37.1%) | 763 (40.6%) |
| Unknown or Declined | 269 (3.2%) | 67 (3.6%) |
| **Language, n (%)** |  |  |
| English | 6,450 (76.2%) | 1,470 (78.1%) |
| Spanish | 1,589 (18.8%) | 335 (17.8%) |
| Other | 424 (5.0%) | 76 (4.0%) |
| **Interpreter Needed, n (%)** |  |  |
| No | 6,645 (78.5%) | 1,512 (80.4%) |
| Yes | 1,818 (21.5%) | 369 (19.6%) |
| **Disposition, n (%)** |  |  |
| Discharge | 7,102 (83.9%) | 1,686 (89.6%) |
| Admit | 1,266 (15.0%) | 183 (9.7%) |
| Transfer to other facility | 52 (0.6%) | 1 (0.1%) |
| Other or Expired | 43 (0.5%) | 11 (0.6%) |
| **Means of Arrival, n (%)** |  |  |
| Self-Arrival | 7,773 (91.8%) | 1,810 (96.2%) |
| Ambulance | 682 (8.1%) | 70 (3.7%) |
| Other | 8 (0.1%) | 1 (0.1%) |

**Summary:** The two groups are well-balanced on patient demographics. Sex (≈54–56% female), language (≈76–78% English), and interpreter need (≈20–22%) differ by less than two percentage points. Racial and ethnic distributions also closely match, with at most a 4-percentage-point absolute difference in any single category. The largest group differences are in disposition (ambient encounters are more likely to end in discharge: 89.6% vs 83.9%) and means of arrival (ambient encounters are more often self-arrivals: 96.2% vs 91.8%). These differences are consistent with the higher concentration of ambient encounters in the Vertical Care and Telemedicine zones (Section 4.4), which preferentially see lower-acuity, ambulatory patients. Disposition and means-of-arrival categories follow the chart-review schema applied uniformly across both groups; left-without-being-seen, eloped, and arrived-in-error encounters were excluded at the cohort-construction stage and do not appear in this table.

## 6.2 Sensitivity Analysis: Adjustment for Patient Demographics

**Model Specification:**

Documentation Time ~ Ambient Scribe + Age + ESI + Zone + Diagnoses +

Sex + Language + Race + Ethnicity + Interpreter +

Zone × ESI + (1|Physician)

Where: Sex = 3 levels (Female, Male, Unknown), Female reference; Language = English, Spanish, Other, or Unknown, English reference; Race = 6 levels with the largest collapsed bucket as reference; Ethnicity = Hispanic or Latino, Non-Hispanic, or Declined or Unknown, reference Declined or Unknown; Interpreter = binary indicator (Yes vs No).

### Table S7. Demographic-Adjusted Sensitivity Results

| **Parameter** | **Primary Model** | **With Demographics** | **Change** |
| --- | --- | --- | --- |
| **Primary Effect** |  |  |  |
| Ambient AI Scribe | −72.6s [−81.4, −63.8] | −71.7s [−80.5, −62.9] | −0.9s (−1.3%) |
| P-value | <.001 | <.001 | – |
| **Demographic Effects** (vs reference, in seconds) |  |  |  |
| Male sex (vs Female) | – | −2.8 [−8.3, +2.6] | P=.31 |
| Sex Unknown (vs Female) | – | +159.7 [−1.0, +320.5] | P=.05 |
| Spanish language (vs English) | – | −3.2 [−21.8, +15.4] | P=.74 |
| Other language (vs English) | – | +5.9 [−13.8, +25.6] | P=.56 |
| Hispanic or Latino ethnicity | – | −11.4 [−27.3, +4.6] | P=.16 |
| Non-Hispanic ethnicity | – | −18.5 [−34.9, −2.1] | P=.027 |
| White race | – | −0.2 [−44.5, +44.1] | P=.99 |
| Asian race | – | +0.8 [−43.9, +45.4] | P=.97 |
| Black race | – | +7.5 [−38.0, +53.0] | P=.75 |
| Pacific Islander race | – | +16.6 [−30.9, +64.2] | P=.49 |
| Other, Declined, or Unknown race | – | −10.9 [−55.3, +33.5] | P=.63 |
| Interpreter needed | – | +17.3 [−0.8, +35.4] | P=.06 |
| **Model Fit** |  |  |  |
| Conditional R² | 0.426 | 0.427 | +0.001 |

**Interpretation:**

1. **Primary effect unchanged.** The ambient AI scribe effect estimate changed by 0.9 seconds (1.3%) when demographics were added — well below any meaningful clinical threshold and consistent with negligible confounding by these characteristics.

2. **One demographic predictor reaches conventional significance.** Encounters classified as Non-Hispanic or Non-Latino are associated with approximately 19 fewer seconds of on-shift documentation than the Unknown or Declined reference category (P=.027). The encounters with an unknown or unrecorded sex category yields a large positive point estimate (+160s) but with a 95% CI that crosses zero (P=.05) and an extremely small n (n = 3, all in the nonambient group), so the apparent signal is best interpreted as encounter-level data quality rather than a meaningful clinical effect. The interpreter-needed indicator shows a borderline positive coefficient (+17s, P=.06) consistent with the additional documentation burden of language-discordant care.

3. **No race effect.** All individual race categories yielded coefficients within ±20 seconds and P-values >.49, indicating no detectable differential physician documentation behavior by patient race in this cohort.

4. **Model fit unchanged.** Conditional R² changed by 0.001 with demographics included, confirming that these variables explain minimal additional variance in physician on-shift documentation time beyond the clinical and workflow factors already in the primary model.

5. **Conclusion.** Patient demographics do not confound the relationship between ambient AI scribe use and documentation time. The primary analysis appropriately controls for clinical and workflow factors (acuity, complexity, zone) without requiring demographic adjustment.

# 7. Software and Computation

**Statistical Software:** Python 3.9; pandas, statsmodels, scipy, numpy, scikit-learn (latest stable versions at the time of analysis).

**Model Estimation:** Linear mixed-effects models: statsmodels.MixedLM. Gamma generalized estimating equations: statsmodels.GEE with Gamma(link=log) family. Convergence: BFGS optimizer with default tolerances; all models converged without warnings.

**Computational Environment:** Apple Silicon workstation, ≥32 GB RAM, macOS.

**Reproducibility:** All analysis code, the BigQuery SQL pipeline, the extracted analytical CSV, and the JSON-formatted output for the primary, secondary, heterogeneity, and robustness models are archived in the project repository. Random seed 42 was used for all stochastic procedures (placebo permutations, propensity-score model resampling).
